# *In vivo* Site-Specific Transfection of Naked Plasmid DNA and siRNAs in Mice by Using a Tissue Suction Device

**DOI:** 10.1371/journal.pone.0041319

**Published:** 2012-07-23

**Authors:** Kazunori Shimizu, Shigeru Kawakami, Kouji Hayashi, Hideyuki Kinoshita, Koichiro Kuwahara, Kazuwa Nakao, Mitsuru Hashida, Satoshi Konishi

**Affiliations:** 1 Institute for Innovative NanoBio Drug Discovery and Development, Graduate School of Pharmaceutical Sciences, Kyoto University, Kyoto, Japan; 2 Ritsumeikan-Global Innovation Research Organization, Ritsumeikan University, Kusatsu, Shiga, Japan; 3 Department of Drug Delivery Research, Graduate School of Pharmaceutical Sciences, Kyoto University, Kyoto, Japan; 4 Department of Medicine and Clinical Science, School of Medicine, Kyoto University Graduate, Kyoto, Japan; 5 Institute for Integrated Cell-Material Sciences, Kyoto University, Kyoto, Japan; 6 Department of Micro System Technology, Ritsumeikan University, Kusatsu, Shiga, Japan; Montana State University, United States of Amercia

## Abstract

We have developed an *in vivo* transfection method for naked plasmid DNA (pDNA) and siRNA in mice by using a tissue suction device. The target tissue was suctioned by a device made of polydimethylsiloxane (PDMS) following the intravenous injection of naked pDNA or siRNA. Transfection of pDNA encoding luciferase was achieved by the suction of the kidney, liver, spleen, and heart, but not the duodenum, skeletal muscle, or stomach. Luciferase expression was specifically observed at the suctioned region of the tissue, and the highest luciferase expression was detected at the surface of the tissue (0.12±0.03 ng/mg protein in mice liver). Luciferase expression levels in the whole liver increased linearly with an increase in the number of times the liver was suctioned. Transfection of siRNA targeting glyceraldehyde 3-phosphate dehydrogenase (GAPDH) gene significantly suppressed the expression of GAPDH mRNA in the liver. Histological analysis shows that severe damage was not observed in the suctioned livers. Since the suction device can be mounted onto the head of the endoscope, this method is a minimally invasive. These results indicate that the *in vivo* transfection method developed in this study will be a viable approach for biological research and therapies using nucleic acids.

## Introduction

In the post-genomic era, increased importance has been placed on the development of *in vivo* transfection techniques that can be used for biological research or gene therapy. Many *in vivo* transfection methods have been developed using recombinant viral vectors or non-viral carriers such as cationic liposomes and polymers [1]–[4]. However, transfection methods for naked nucleic acids, including plasmid DNA (pDNA) or siRNA, have many advantages, including convenient preparation, ease of handling, and lack of toxicity associated with the transfection agents. Therefore, this is largely considered to be the simplest and safest method [5].

Liu *et al.* reported that non-invasive gene delivery to the liver was performed by a mechanical massage around the abdomen after intravenous injection of naked pDNA in mice [6], [7]. Inspired by their study, our group found that direct pressure to the kidneys, spleen, and liver induces the transfection of naked nucleic acids, which we termed as tissue pressure-mediated transfection [8]–[10]. This method has been used with naked pDNA, siRNA, and microRNA [8]–[11], and the miR-200 family of microRNAs introduced by renal pressure-mediated transfection ameliorated renal tubulointerstitial fibrosis in mice [11]. Further, we previously reported that the secretion of pro-inflammatory cytokines was not observed under the experimental conditions for transfection and the degree of direct pressure applied to the target tissue is one of the key factors for controlling the expression levels of the transfected pDNA [9].

In tissue pressure-mediated transfection, 2 objects were used to apply direct pressure effectively to the target tissue; the first is used to directly press the target tissue, and the other is used to support the pressed tissue. Examples include the index finger and the thumb [8], [11], a syringe-modified pressure controlling device and a spatula [9], [10], and a pneumatic balloon actuator and a renal case [12]. We have used these objects to perform tissue pressure-mediated transfection in small animals such as mice and rats. However, considering future clinical use, we sought to develop an easier method using a simpler device that required minimally invasive treatment. Previously, we developed a micro-pneumatic suction device for medical diagnosis and operation [13]. The simple suction device was used to fix medical Micro Electro Mechanical Systems (MEMS) such as temperature sensors or micropumps on the surface of pulsating target tissues. The tissue suction device, made of polydimethylsiloxane (PDMS), suctions a tissue surface by applying negative pressure. Although the suctioned part of the tissue was deformed temporarily, *in vivo* experiments revealed that the damage was negligible [13]. Furthermore, it has been demonstrated that the suction device can be mounted to the head of the endoscope, allowing for increased use in a clinical setting [13].

Our previous results using the pneumatic balloon actuator and the renal case suggested that tissue deformation following the tissue pressure would be a key factor for the transfection efficiency of naked pDNA [12]. This prompted us to investigate whether a tissue suction device could be used for *in vivo* site-specific transfection of naked pDNA or siRNA in mice. In this study, transfection of mouse kidney, liver, spleen, heart, duodenum, skeletal muscle, and stomach were evaluated after tissue suction by a PDMS device following intravenous injection of naked pDNA or siRNA. This is our initial study concerning *in vivo* site-specific transfection using a tissue suction device.

## Results

### Naked pDNA Transfection by Tissue Suction

**Figure 1:**
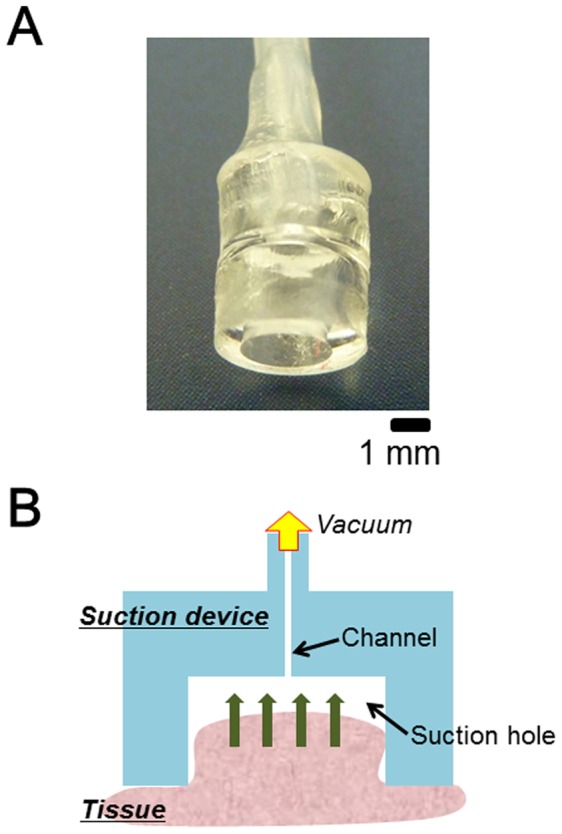
Tissue suction by using the device. A) Representative photograph of the tissue suction device (type II). B) Schematic illustration of *in vivo* transfection by tissue suction. The surface of the target tissues was suctioned by the suction device just after intravenous injection of naked nucleic acids.

**Figure 2:**
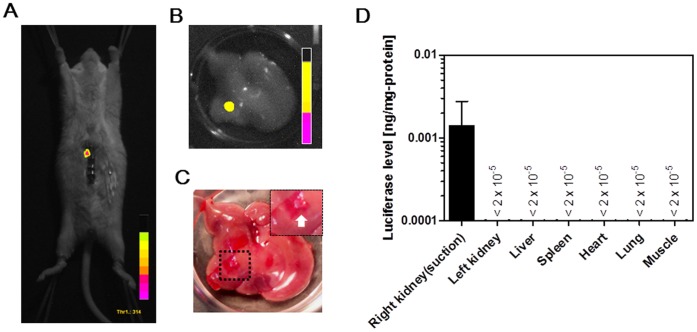
*In vivo* transfection of naked pDNA by tissue suction. A) *In vivo* imaging of luciferase activity in a mouse liver that was suctioned once by the type I device just after intravenous injection of pCMV-Luc. B) *Ex vivo* imaging of luciferase activity in the liver suctioned by the type I device. C) Bright field image of (B). D) Luciferase levels of various tissues. The right kidney in mice was suctioned once by the type III device. Each value represents means + SD (n  = 4). All mice were alive at the end of the experiment.

To prove our hypothesis, mouse livers were transfected with pCMV-Luc by using the tissue suction devices (Fig. 1). The liver surface of the anesthetized mice was suctioned once immediately after intravenous injection of pCMV-Luc, and *in vivo* imaging of luciferase activity was performed 6 h after the suction. As shown in Fig. 2A, luciferase expression was detected at the region where the liver had been suctioned. *Ex vivo* imaging of the suctioned liver clearly shows that the luciferase was expressed at the site of tissue suction (Fig. 2B and 2C). The tissue suction device was also used to transfect pDNA into mouse kidneys. The right kidney of the anesthetized mouse was suctioned by the suction device just after pCMV-Luc injection, and the luciferase levels in various tissues were investigated. As shown in Fig. 2D, luciferase expression was specifically found in the suctioned right kidney. Therefore, as we expected, the transfection of naked pDNA was possible by using the tissue suction device.

### Investigation of Applicable Tissues of pDNA Transfection by Tissue Suction

**Figure 3:**
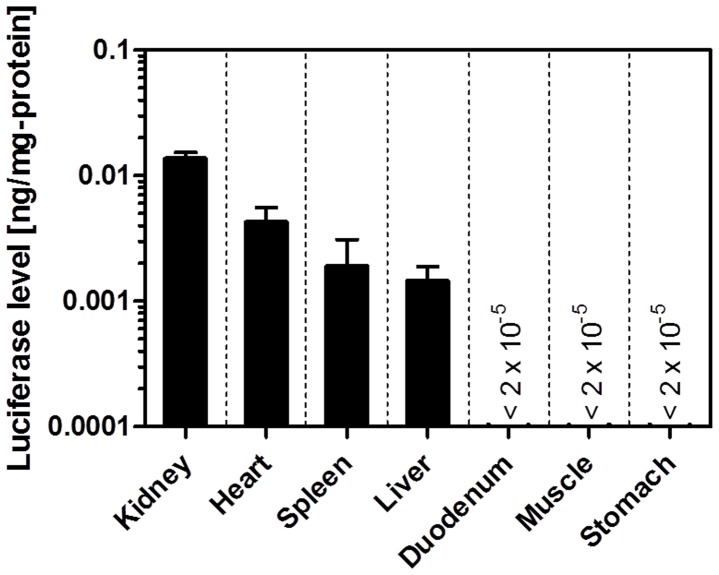
*In vivo* transfection to various tissues by tissue suction. *In vivo* transfection by tissue suction was applied to various tissues (including the kidney, heart, spleen, liver, duodenum, muscle, and stomach). A type III device was used for the muscle and stomach. A type IV device was used for the kidney, heart, spleen, liver, and duodenum. Each value represents means + SD (n  = 3 [the kidney, spleen, and muscle], n  = 4 [the liver, duodenum, and stomach], or n  = 5 [the heart]). All mice were alive at the end of the experiment.

Next, we investigated the extended application of the naked pDNA transfection by tissue suction. Luciferase activity of the tissues, including the kidney, liver, heart, spleen, duodenum, muscle, and stomach, were examined 6 h after the mice had received an intravenous injection of pCMV-Luc, immediately followed by suction of each tissue. As shown in Fig. 3, high luciferase gene expression was obtained in the right kidney, heart, spleen, and liver. The expression levels were approximately 0.014 ng/mg protein for the right kidney, 0.006 ng/mg protein for the heart, 0.002 ng/mg protein for the spleen, and 0.001 ng/mg protein for the liver. In contrast, the luciferase levels of the duodenum, muscle, and stomach were less than 2×10^−5^ ng/mg protein.

### Distribution of Transgene Activities

**Figure 4:**
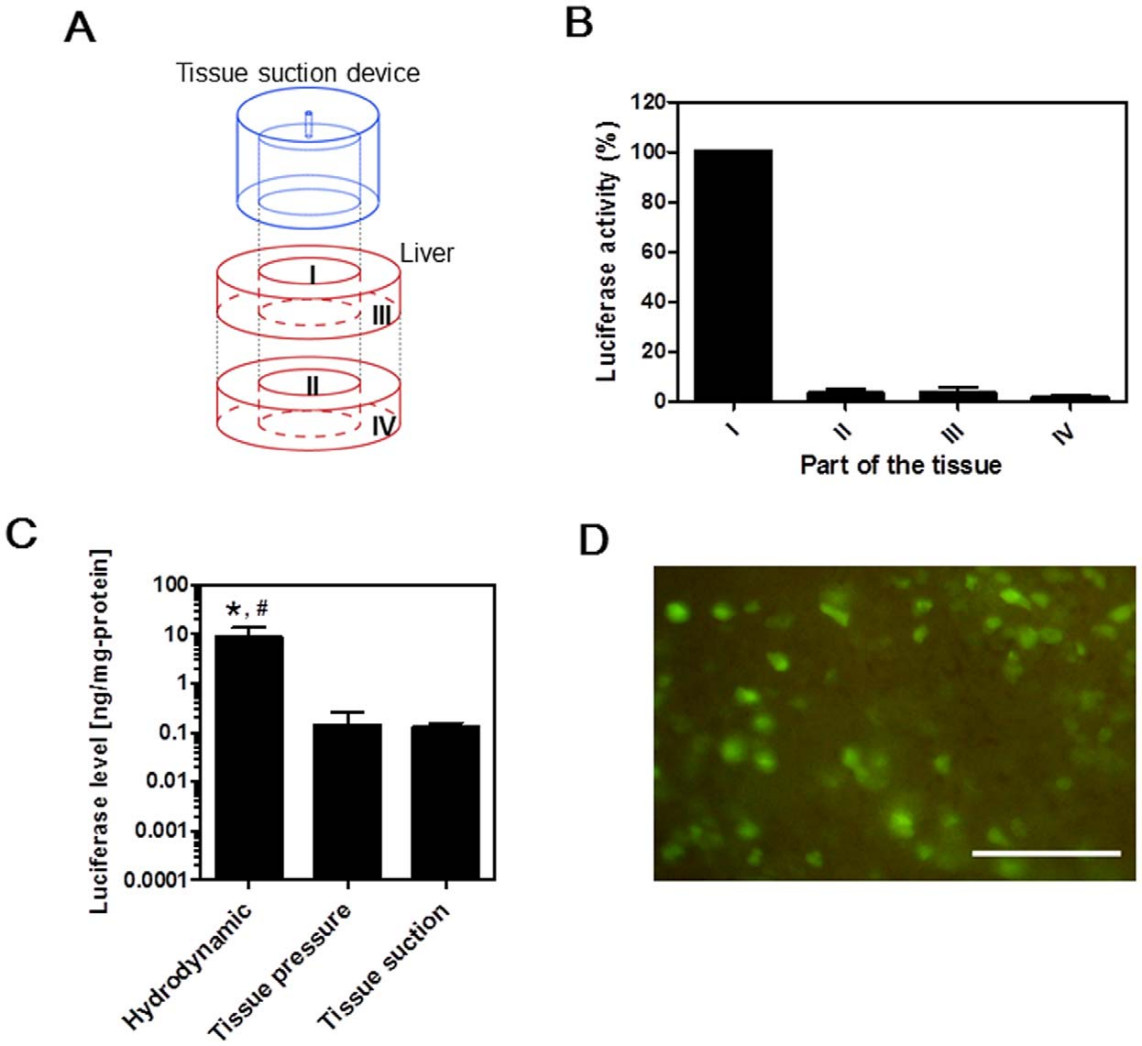
Luciferase gene expression around the suctioned region of the liver. A) Schematic illustration of the experiment. The liver was suctioned by the type III device, and the liver was cut into 4 parts (I to IV). B) Luciferase levels in each of the 4 parts. The values were normalized to levels from Part I (n  = 4). C) The luciferase level of *in vivo* transfection by tissue suction was compared with that by hydrodynamic method and tissue pressure-mediated transfection. *p<0.01 versus tissue pressure. ^#^p<0.01 versus tissue suction. Each value represents means + SD (n  = 3 [hydrodynamic method], n  = 8 [tissue pressure], or n  = 5 [tissue suction]). D) Imaging of the top surface of Part I transfected with pCMV-GFP. Scale bar, 200 µm. All mice were alive at the end of the experiment.

The distribution of luciferase gene expression around the suctioned region of the liver was investigated. The liver surface of the anesthetized mice was suctioned once just after intravenous injection of pCMV-Luc. Six hours after suction, the tissue was separated into 4 parts as shown in Fig. 4A, and the luciferase level of each part was independently examined (Fig. 4A). The highest luciferase gene expression was obtained from Part I, and this level was set at 100% (Fig. 4B). In contrast, the relative luciferase expression levels were approximately 3.2% for Part II, 3.4% for part III, and 1.5% for Part IV. Subsequently, the luciferase expression induced by tissue suction was compared to that of other methodologies such as the hydrodynamic method and the liver pressure-mediated transfection (Fig. 4C). For transfection by tissue suction, the luciferase level at Part I was determined to be 0.12±0.03 ng/mg protein. For the hydrodynamic method, the luciferase level reached a higher level of 6.29±5.98 ng/mg protein. For the liver pressure-mediated transfection method, a luciferase level of 0.14±0.12 ng/mg protein was obtained at the treated part, and this was not significantly different from the results obtained by tissue suction. Furthermore, the distribution of the liver cells transfected by tissue suction was investigated at the cellular level using pCMV-green fluorescent protein (GFP). A large number of GFP-expressing cells were observed at the top surface of Part I (Fig. 4D).

### Effects of the Number of Tissue Suctions on Luciferase Expression

**Figure 5:**
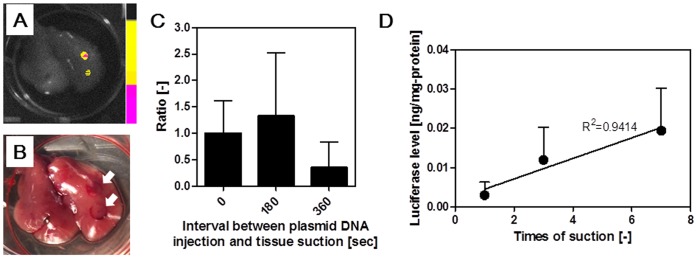
Effects of the number of tissue suctions on luciferase levels. A) *Ex vivo* imaging of luciferase activity in the liver simultaneously suctioned at 2 different parts. Two type III devices were used. B) Bright field image of (A). C) Effects of interval between pCMV-Luc injection and tissue suction on the luciferase levels in liver. The liver was suctioned by type III devices. D) Effects of the number of tissue suctions on luciferase levels were investigated. Liver were serially suctioned 1, 3, and 7 times by using the type III device within 180 s of pCMV-Luc injection. All mice were alive at the end of the experiment.

We investigated the effects of the number of tissue suctions on pDNA expression. First, 2 different parts of the tissue were simultaneously suctioned by using 2 tissue suction devices just after intravenous injection of pCMV-Luc. *In vivo* imaging of luciferase activity was then performed 6 h after the suction. As shown in Fig. 5A and 5B, luciferase was expressed at the 2 different regions where the liver was simultaneously suctioned. Next, a different part of the tissue was serially suctioned. Since luciferase expression levels did not decrease if the liver was suctioned after 180 s following pCMV-Luc injection (Fig. 5C), the liver surface was suctioned 1, 3, or 7 times within 180 s. As shown in Fig. 5D, the luciferase level in the whole liver increased linearly (R^2^ = 0.9414), with an increase in the suction number and reached approximately 0.02 ng/mg protein.

### siRNA Transfection of the Liver by Tissue Suction

**Figure 6:**
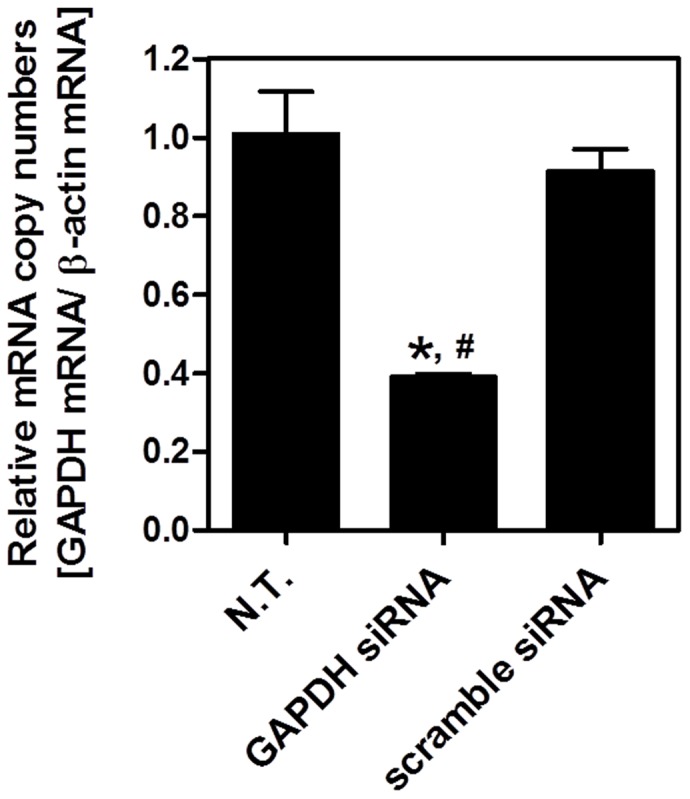
siRNA transfection to the liver by tissue suction. GAPDH siRNA and scramble siRNA were transfected by using type II device. mRNA expression of GAPDH after 24 h of transfection was measured. Each value represents means + SD (n  = 3 [N.T.], n  = 4 [GAPDH siRNA and scramble siRNA]). There was a statistically significant difference between 3 groups (ANOVA; F  = 99.72, p<0.0001). Post-hoc analysis (Bonferroni’s test) was performed. *p<0.001 versus N.T. ^#^p<0.001 versus scramble siRNA. All mice were alive at the end of the experiment.

To evaluate the potential for naked oligonucleotide delivery of the transfection by tissue suction, we demonstrated silencing of an endogenous gene, glyceraldehyde 3-phosphate dehydrogenase (GAPDH) [14], [15]. Transfection of GAPDH siRNA was performed on mouse livers by using the tissue suction device. The liver surface of the anesthetized mice was suctioned once just after the administration of GAPDH siRNA, and the GAPDH mRNA expression of suctioned part of the liver (Part I in Fig. 4A) was investigated 24 h after suction. As shown in Fig. 6, the GAPDH mRNA expression in the liver was significantly reduced, with about 61% suppression. In contrast, when the scramble siRNA were administered, no marked suppression of GAPDH mRNA level was observed (Fig. 6).

### Effects of Tissue Suction on Hepatic Toxicity

**Figure 7:**
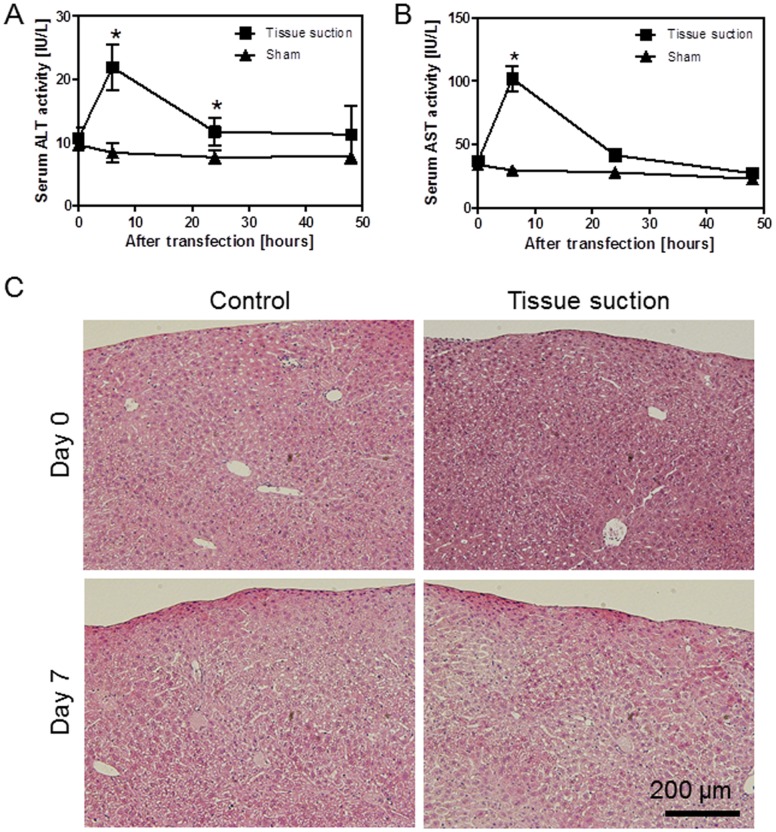
Effects of tissue suction on hepatic toxicity. A) Alanine aminotransferase (ALT) in serum. ALT activity was measured at 0, 6, 24, and 48 h after transfection. Type III device was used. Each value represents mean ± SD (n  = 3 [sham operation], or n  = 4 [tissue suction]). *p<0.05 versus sham operation. B) Aspartate aminotransferase (AST) in serum. AST activity was measured at 0, 6, 24, and 48 h after transfection. Type III device was used. Each value represents mean ± SD (n  = 3 [sham operation], or n  = 4 [tissue suction]). *p<0.05 versus sham operation. C) HE staining of the liver section. The suctioned part of the liver (Part I in Fig. 4A) was sampled at 0 and 7 days after tissue suction. Type III device was used. All mice were alive at the end of the experiment.

Alanine aminotransferase (ALT) and aspartate aminotransferase (AST) activities in the serum were examined to determine the occurrence of liver toxicity after treatment with the tissue suction device (Fig. 7A and 7B). pCMV-Luc was intravenously injected, and the liver surface was suctioned once by the device. After 0, 6, 24, and 48 h, ALT and AST activities in the serum were measured. ALT activity at 6 and 24 h and AST activity at 6 h in the mice treated with the tissue suction device were significantly higher than those in mice with the sham operation (p<0.05); however, the activities returned to normal levels within 48 h (Fig. 7A and 7B). We also performed HE staining of the liver sections to examine damage that may be caused by liver suction. However, we did not observe any severe damage to the suctioned livers following transfection (Fig. 7C).

## Discussion

In the present study, it was shown that *in vivo* transfection of naked nucleic acids such as pDNA and siRNA can be achieved by tissue suction. Previously, positive pressure had been used to directly press the target tissue to induce transfection [8]–[12]. The present study demonstrates the first use of negative pressure to induce transfection. The negative pressure supplied by the tissue suction device deformed the target tissue and induced transfection. Transfection by negative pressure has 2 main advantages over positive pressure: (i) it is easier to fix the relative position between the target tissue and the device with negative pressure [13], [16] and (ii) the tissue suction devices are simpler than the combinations of 2 objects used to press tissues in the positive pressure studies [8]–[12]. Therefore, our findings suggest that it is now possible to perform tissue pressure-mediated *in vivo* transfection with greater ease than previously reported. Moreover, since the tissue suction devices are small enough to be mounted to the head of the endoscope, it is possible to perform transfections less invasively than before. Thus, we have successfully innovated a useful procedure for tissue pressure-mediated transfection employing tissue suction.

We made the tissue suction devices in several sizes (Table 1) and suctioned the tissue surface until the inner space of the suction devices was filled with deformed tissue. In this study, we selected the device type according to tissue size or surgical procedure. For the smaller tissues including the spleen and duodenum, we used the device with the smaller inner diameter (type IV). The type IV device was also used to suction the heart due to limited space between the ribs. We could use all the types of suction devices listed in Table 1 for larger tissues including the liver and found that they could induce pDNA transfection. However, our preliminary data showed that device inner diameter and height affected transfection efficiency (Fig. S1). Furthermore, hemorrhage was occasionally observed around the suctioned parts of the tissue (around part III in Fig. 4A) when the target tissue was excessively deformed by high negative pressure suction. Thus, further studies should be conducted to optimize the size of the tissue suction device and the amount of negative pressure applied to the target tissues. Data from such experiments could allow researchers to effectively perform the tissue suction method without causing toxicity or damage to the target tissues.

**Table 1:** Tissue suction devices used in this study.

Type	Inner diameter [mm]	Outer diameter [mm]	Height [mm]	Applied tissues
I	4	8	3	Liver
II	4	6	3	Liver, kidney
III	3	5	3	Liver, kidney, muscle, stomach
IV	2	3	3	Liver, kidney, heart, spleen, duodenum

*In vivo* transfection by tissue suction achieved site-specific gene transfection in the applied organs (Figs. 2, 4, and 5). In contrast, conventional methods, including recombinant viral vectors and non-viral carriers, have difficulty in selectively inducing transfection within specific target tissues. However, selective hydrodynamic gene delivery of naked pDNA to the liver segments by using balloon catheters was recently performed [17], [18]. The catheter-based method is a promising technique for clinical use, but requires specialized skills and may be difficult to apply to relatively small animals. In contrast, transfection by tissue suction is easy to perform and can be applied to both large and small animals. Transfection can also be induced at a specific site of the tissue; therefore, it represents a unique technology for performing selective *in vivo* transfection.

Transfection by tissue suction can control the amount of transgene expressed in the target tissue (Fig. 5); our experiments demonstrated that expression levels of the transfected gene in the whole liver increased linearly with an increase in the number of liver suctions (Fig. 5D). Therefore, it is expected that the concentration of the expressed protein in the serum can also be controlled by using the pDNA that encodes secretory proteins. We believe that this ability to control is especially valuable when transfecting genes that may have toxicity at high concentrations.

On the other hand, it is one of the strategies that uses pDNA to encode non-secretory protein or the oligonucleotides for silencing gene expression. For this strategy, it is important to consider the number of transfected cells within the suctioned part of the tissue. In the present study, naked pCMV-GFP was transfected to the liver via tissue suction, and a large number of GFP-expressing cells were observed at the top surface of the suctioned part of the liver (Fig. 4D). Moreover, silencing of the expression of an endogenous gene, GAPDH, within the suctioned part of the liver was demonstrated by transfection of GAPDH siRNA (Fig. 6). The earlier study showed that the renal pressure-mediated transfection method could transfect the microRNA precursor into many tubular cells in the kidney and ameliorate renal tubulointerstitial fibrosis [11]. Thus, it is expected that transfection by tissue suction is applicable for expressing non-secretory protein using pDNA or silencing gene expression using siRNA or microRNA. Nonetheless, a detailed consideration should be performed for further applications of this method.

We found that transfection by tissue suction was effective in the kidney, liver, spleen, and heart, although not the duodenum, muscle, or stomach (Fig. 3). In the previous tissue pressure-mediated transfection study, however, it was reported that the transfection of naked pDNA was possible in the kidney, liver, and spleen, but not the heart [9]. This may be attributed to the fact that the heart was not directly pressed for heart pressure-mediated transfection; the abdomen and chest of the mice were held between the thumb and the index and middle fingers of both hands, and pressed without exposure of the tissues [9]. In the present study, the heart was suctioned directly by using the tissue suction device and we found that naked pDNA transfection could be achieved in the heart. This is the first study that demonstrates *in vivo* transfection by tissue suction in the heart. These findings indicate that heart gene therapy is a promising application of transfection by tissue suction. So far, several methods that can induce transfection of naked pDNA into cardiac tissue have been reported. Direct myocardial injection of naked pDNA has been used for gene therapy for heart failure [19], [20]; retrograde injection of naked pDNA in the coronary sinus by catheterization was used for whole cardiac gene transfer [21], and *in vivo* electroporation was conducted on rat and porcine hearts [22], [23]. As shown in Fig. 4C, the transfection efficiency by tissue suction in the liver was equal to that of the liver pressure-mediated transfection method. We previously compared the efficiency of the kidney pressure-mediated transfection method with that of other transfection methods–renal parenchymal injection and a combination method with electroporation in the kidney–and reported almost equal efficiency [8]. Considering these results, we expected both the heart suction method and the other methods to be simple and promising techniques for heart gene therapy [19]–[23].

For potential clinical use, it is important to consider the toxicity of transfection by tissue suction on the treated tissues. In the present study, we performed both biochemical and histological assays to assess the toxicity of transfection by tissue suction of the liver (Fig. 7). The ALT and AST activities were transiently increased and returned to normal levels within a few days (Fig. 7A and 7B), which is in good agreement with the time-course profiles of ALT and AST activities of the hydrodynamic method [24], [25]. It was also reported previously that renal pressure-mediated transfection did not induce pro-inflammatory cytokines in the serum and did not affect renal function [8], [9]. Considering these results, we hypothesized that tissue pressure-mediated transfection, regardless of suction or pressure, is generally safe *in vivo*. Nevertheless, further investigation about the safety of tissue suction methods is warranted.

In conclusion, the present study investigated the feasibility of an *in vivo* transfection method by using a tissue suction device for the transfection of naked pDNA or siRNA in mice, and demonstrated that this method would be an effective approach in biological research and therapies using nucleic acids.

## Materials and Methods

### pDNA, siRNAs, and Mice

pDNA-encoding complementary luciferase DNA (pCMV-Luc) [26] and emerald GFP (pCMV-GFP; pcDNA6.2-EmGFP, Life Technologies, Carlsbad, CA) were used. They were driven by the CMV immediate-early promoter. The amplification, isolation, and purification of pDNA were performed as described previously [26]. siRNAs (21 mer) with 3′-dTdT overhangs were chemically synthesized by SIGMA Aldrich Japan (Hokkaido, Japan), and siRNA sequences are shown as follows: mouse GAPDH siRNA, 5′-CAA GAG AGG CCC UAU CCC AdTdT-3′ (sense) and 5′-UGG GAU AGG GCC UCU CUU GdTdT-3′ (antisense); scrambled siRNA, 5′-CGC AAC UAC CGA UGC GAA CdTdT-3′ (sense) and 5′-GUU CGC AUC GGU AGU UGC GdTdT-3′ (antisense). ICR mice (female, 5 weeks old) were purchased from Japan SLC Inc. (Shizuoka, Japan). All animal experiments were carried out in accordance with the Guide for the Care and Use of Laboratory Animals, as adopted and promulgated by the U.S. National Institutes of Health (Bethesda, MD) and the Guidelines for Animal Experiments of Kyoto University (Kyoto, Japan). The study protocols permission numbers 2010–47, 2011–39, and 2012–50 were approved by the Animal Research Committee, Kyoto University.

### Fabrication of Tissue Suction Devices

A tissue suction device was fabricated as described previously, with some modifications [13]. Briefly, a polydimethylsiloxane (PDMS) (10∶1) solution was poured into a plastic dish and cured at 75°C for 2 h. The PDMS slab was punched out using a disposable biopsy punch (Kai Industries Co., Ltd., Gifu, Japan), and ring-shaped PDMS structures were fabricated. Then, the ring-shaped structures were bonded to disc-shaped PDMS structure with the same diameter, and the disc-shaped part was punched out to connect a silicone tube with an outer diameter of 1 mm used for supplying negative pressure. Various suction device specifications such as size and shape can be designed depending on the requirements [13]. Four sizes of the device structure were used in the present study (Fig. 1 and Table 1).

### pDNA and siRNA Transfection Using Tissue Suction Devices

In a typical case of pDNA transfection using tissue suction devices, the mice were anesthetized with isoflurane, and the target tissue was minimally exposed. pCMV-Luc (100 µg in 200 µL of saline) was intravenously injected into the mice, and the target tissue was suctioned by the device with small amounts of negative pressure (Fig. 1). The tissue was suctioned until the inner space of the device was filled with the deformed tissue. In case of the kidney, liver, spleen, stomach, and duodenum, the target tissue was slightly exposed by a midline incision, and the target part of the tissue was suctioned by the device. In case of the heart, anesthetized mice were ventilated by a respirator (SN-480-7; Shinano, Tokyo, Japan) during the treatment. The left costal cartilage (the fourth rib) was removed to minimally expose the left ventricle, and the ventricle was suctioned by the device. In case of muscle, the dermis of the left hind leg was cut to expose the femoral muscle, and the muscle was suctioned by the device.

In siRNA transfection experiments, anesthetized mice were intravenously injected with GAPDH siRNA or scramble siRNA (50 µg in 200 µL saline), immediately followed by small negative pressure-mediated suctioning of the liver by the suction device.

### pDNA Transfection by the Hydrodynamic Method

The hydrodynamic method was performed as described previously [24]. Five micrograms of pCMV-Luc in 1600 µL of saline was intravenously injected within 5 s.

### pDNA Transfection by the Liver Pressure-mediated Transfection Method

The liver pressure-mediated transfection method was performed as previously described [9]. The applied pressure was controlled at around 0.5 N/cm^2^ by using the pressure control devices, namely, a syringe-like device and a spatula.

### Luciferase activity Assay and Imaging

Gene expression levels were determined by luciferase assay after 6 h of tissue suction as described previously [26]. Imaging of luciferase activity was performed following a previously described method [8].

### Imaging of the GFP-expressing Cells

pCMV-GFP (500 µg in 200 µL of saline) was intravenously injected into the mice and the livers were suctioned using the type III device. After 6 hours, the mice were sacrificed and the surfaces of the target liver parts were observed under an inverted fluorescence microscope (BZ-8100, Keyence, Osaka, Japan).

### Quantitative Real-time RT-PCR

Total RNA was isolated from the cells and organs using a GenElute Mammalian Total RNA Miniprep Kit (Sigma- Aldrich). Reverse transcription of mRNA was carried out using PrimeScript® RT reagent Kit (Takara Bio, Shiga, Japan). The detection of complementary DNA (GAPDH and β-actin) was conducted using real-time PCR using SYBR® Premix Ex Taq (Takara Bio) and a Lightcycler Quick System 350S (Roche Diagnostics, Indianapolis, IN). Primers for GAPDH and β-actin cDNA were synthesized by Invitrogen as follows: GAPDH, 5′-CTC ACT CAA GAT TGT CAG CAA TG-3′ (forward) and 5′-GGC AGT GAT GGC ATG GAC TGT-3′ (reverse); β-actin, 5′-GTT CTA CAA ATG TGG CTG AGG ACT T-3′ (forward) and 5′-TTG GGA GGG TGA GGG ACT T-3′ (reverse). mRNA copy number was calculated for each sample from the standard curve using the thermal-cycler software (‘Arithmetic Fit Point analysis’ for the Lightcycler). Results were expressed as relative copy number calculated relative to β-actin mRNA (GAPDH mRNA copy number/β-actin mRNA copy number).

### Measurement of the Transaminase Activity in Serum

Alanine aminotransferase (ALT) and aspartate aminotransferase (AST) activities in the serum were determined as described previously [27]. Briefly, the serum was collected from the transfected mice 0, 6, 24 and 48 h after transfection. Transaminase CII-Test Wako kit (Wako Pure Chemical Industries, Tokyo, Japan) was used according to the manufacturer’s instructions.

### Hematoxylin and Eosin Staining

The mice livers were harvested at 0 and 7 days after the suction and fixed in 10% neutral buffered formalin. Four-micrometer thick paraffin sections were stained with hematoxylin and eosin (HE). The histology of the liver sections was microscopically examined.

### Statistical Analysis

Prism 5 software (Graphpad Software, La Jolla, CA, USA) was used. Statistical significance was determined by two-tailed t-test or one-way analysis of variance (ANOVA), followed by Bonferroni’s test.

## Supporting Information

Figure S1 **Effects of the size of the tissue suction devices on luciferase levels.** *In vivo* transfection by tissue suction was applied to the right kidney by using type II and IV device. *p<0.05 versus type IV device (n  = 6 [type II], n  = 3 [type IV]). All mice were alive at the end of the experiment.
(TIF)
