# Supplementary figures and images for: In vivo Site-Specific Transfection of Naked Plasmid DNA and siRNAs in Mice by Using a Tissue Suction Device
Source: PLoS One. 2012 Jul 23;7(7):e41319. doi: 10.1371/journal.pone.0041319 (PMC3402481; doi:10.1371/journal.pone.0041319)

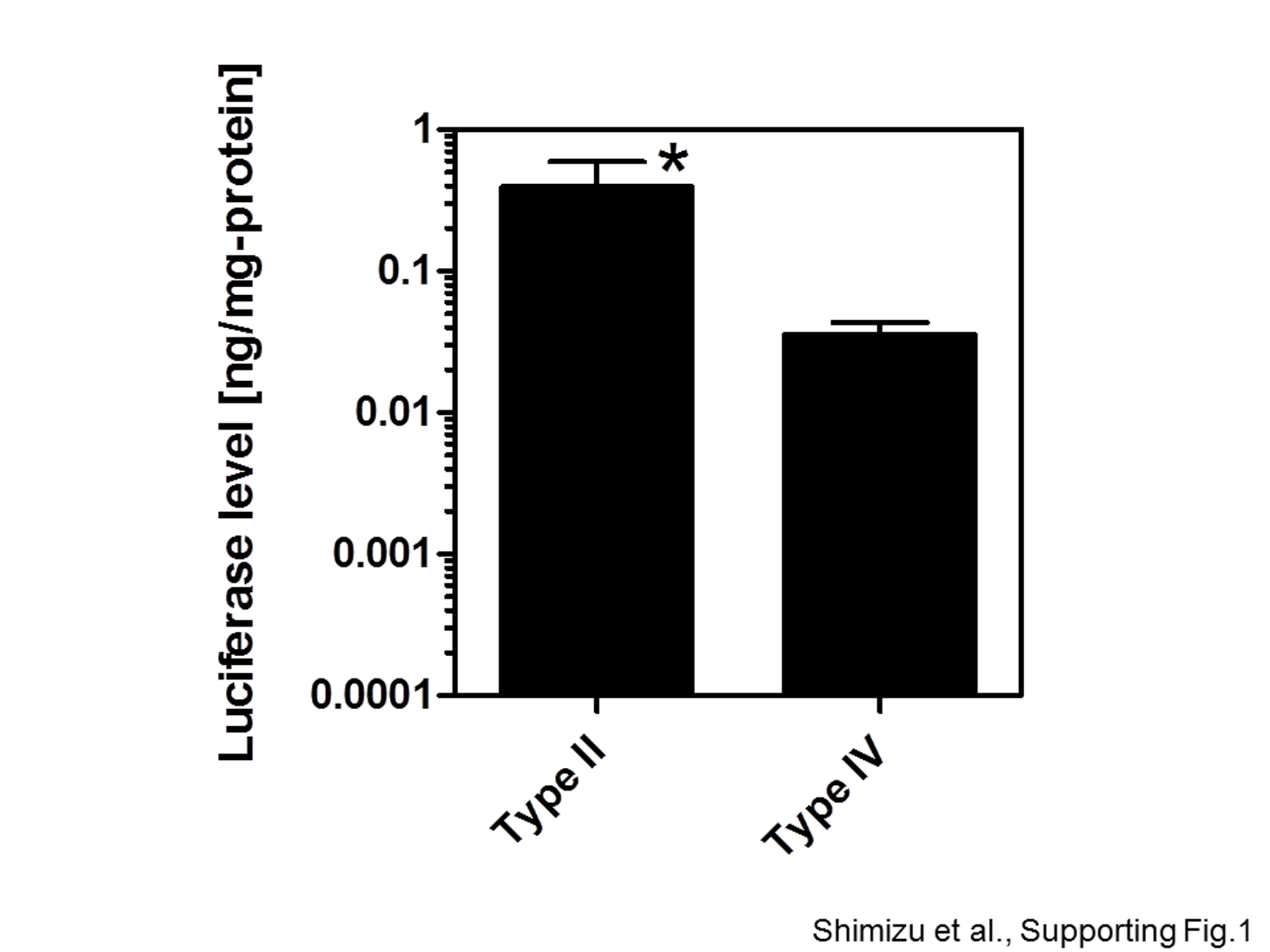

Supplement: Figure S1 — Effects of the size of the tissue suction devices on luciferase levels. In vivo transfection by tissue suction was applied to the right kidney by using type II and IV device. *p<0.05 versus type IV device (n = 6 [type II], n = 3 [type IV]). All mice were alive at the end of the experiment. (TIF) [file pone.0041319.s001.tif]
